# Protein kinases MpkA and SepH transduce crosstalk between CWI and SIN pathways to activate protective hyphal septation under echinocandin cell wall stress

**DOI:** 10.1128/msphere.00641-24

**Published:** 2024-12-13

**Authors:** Alexander G. Doan, Jessica E. Schafer, Casey M. Douglas, Matthew S. Quintanilla, Meredith E. Morse, Harley Edwards, Walker D. Huso, Kelsey J. Gray, JungHun Lee, Joshua K. Dayie, Steven D. Harris, Mark R. Marten

**Affiliations:** 1 Department of Chemical, Biochemical, and Environmental Engineering, University of Maryland, Baltimore County, Baltimore, Maryland, USA; 2 Department of Plant Pathology, Entomology, and Microbiology, Iowa State University, Ames, Iowa, USA; Kyungpook National University, Daegu, South Korea

**Keywords:** cell signaling, gene regulation, MAP kinases, cell wall integrity, septation initiation network, *Aspergillus*, ascomycetes, drug resistance mechanisms, growth on coverslip, hyperseptation, mitotic exit network, filamentous fungi

## Abstract

This study investigates a previously unreported stress signal transduced as crosstalk between the cell wall integrity (CWI) pathway and the septation initiation network (SIN). Echinocandins, which target cell wall synthesis, are widely used to treat mycoses. Their efficacy, however, is species specific. Our findings suggest that this is due largely to CWI–SIN crosstalk and the ability of filamentous species to fortify with septa in response to echinocandin stress. To better understand this crosstalk, we used a microscopy-based assay to measure septum density, aiming to understand the septation response to cell wall stress. The echinocandin micafungin, an inhibitor of β-(1,3)-glucan synthase, was employed to induce this stress. We observed a strong positive correlation between micafungin treatment and septum density in wild-type strains. This finding suggests that CWI activates SIN under cell wall stress, increasing septum density to protect against cell wall failure. More detailed investigations, with targeted knockouts of CWI and SIN signaling proteins, enabled us to identify crosstalk occurring between the CWI kinase, MpkA, and the SIN kinase, SepH. This discovery of the previously unknown crosstalk between the CWI and SIN pathways not only reshapes our understanding of fungal stress responses, but also unveils a promising new target pathway for the development of novel antifungal strategies.

**IMPORTANCE:**

Echinocandin-resistant species pose a major challenge in clinical mycology by rendering one of only four lines of treatment, notably one of the two that are well-tolerated, ineffective in treating systemic mycoses of these species. Previous studies have demonstrated that echinocandins fail against highly polarized fungi because they target only apical septal compartments. It is known that many filamentous species respond to cell wall stress with hyperseptation. In this work, we show that echinocandin resistance hinges on this dynamic response, rather than on innate septation alone. We also describe, for the first time, the signaling pathway used to deploy the hyperseptation response. By disabling this pathway, we were able to render mycelia susceptible to echinocandin stress. This work enhances our microbiological understanding of filamentous fungi and introduces a potential target to overcome echinocandin-resistant species.

## INTRODUCTION

Fungi comprise a diverse group of species capable of living in a wide range of environments, including as pathogens in both animal and plant tissues (1, 2). Notable human pathogens include *Aspergillus fumigatus*, *Candida albicans*, *Cryptococcus* spp., and *Mucor* spp. (3), which are responsible for a majority of the 1.5 million deaths caused by fungal infections annually (4). Understanding the molecular mechanisms that underlie these pathogens' ability to respond to stress is crucial for elucidating their virulence and developing strategies to mitigate their pathogenicity.

To date, there are only four lines of treatment available for systemic mycoses: azoles, echinocandins, amphotericin B, and flucytosine in combination with amphotericin B, all of which target either the fungal cell wall or membrane and have species-specific efficacy (5). The high mortality of systemic mycoses (4), combined with the advancement of drug-resistant strains, presents a dire need to develop new lines of treatment (5).

Although many of the mechanisms we discuss are conserved broadly, for brevity, we use *Aspergillus* names for the genes and proteins discussed in this paper and provide the names of orthologous proteins in Table 1 for the reader to compare with other common ascomycete models.

**TABLE 1:** Orthology of signaling proteins described in this work for common model ascomycetes

*A. nidulans*	*S. pombe*	*S. cerevisiae*	*N. crassa*
AnkA	Wee1	SWE1	STK-19
BckA	Mkh1 (BH)^*a*^	BCK1	MIK-1
BubA	Cdc16	BUB2	DIV-2
ByrA	Byr4		NCU01584
MkkA	Pek1	MKK1, MKK2	MEK-1, MEK-2
MobA	Mob1	MOB1	MOB-1
MpkA	Pmk1, Spk1	SLT2, KSS1, FUS3	MAK-1, MAK-2
NimX	Cdc1/Cdc2, Pef1	CDC28, PHO85	CDC28, MDK-1
PkcA	Pck1, Pck2	PKC1	PKC
PomA	Pom1, Pom2, Ppk15	YAK1	PRK-2, STK-46
RlmA	Mbx2	SMP1	TCF-23
SepH	Cdc7	CDC15	CDC15
SepL	Ppk11, Sid1	SPS1, KIC1	PRK-9, STK-6
SepM	Cdc14		DIV-36
SidB	Sid2	DBF2, DBF20	DBF-2
SpgA	Spg1	TEM1	DIV-17

^*a*^ BH, BlastP best hit.

The fungal cell wall is critical to viability and pathogenicity (6) and is targeted by echinocandins through inhibition of β-(1,3)-glucan synthase (5), which is essential for the formation of the major cell wall structural component β-(1,3)-glucan (6, 7). Fungi are able to respond to echinocandin-induced wall stress through the cell wall integrity (CWI or CWIS) pathway. CWI works to maintain integrity of the cell wall in response to cell wall stress and is highly conserved in the fungal kingdom (7–10). The CWI pathway is activated when wall stress triggers cell surface receptors (11). These receptors activate the guanine nucleotide exchange factor (GEF), Rom2, which activates the guanine nucleotide-binding protein (G-protein), RhoA (9, 12). RhoA then phosphorylates PkcA kinase (11, 13–16), which initiates the CWI mitogen-activated protein kinase (MAPK) cascade BckA-MkkA-MpkA (17–20). MpkA is the terminal kinase of CWI and activates the transcription factor RlmA to regulate cell wall biosynthesis genes transcriptionally (9, 19, 21). MpkA also activates other proteins directly through protein–protein interactions (18, 22).

Filamentous fungi are able to preserve vitality during echinocandin-induced wall stress through the construction of septa (23–25), which are structures used to partition hyphae into multiple compartments (26–34). The compartmentalization of hyphal cytoplasm by septa protects germlings from a complete loss of cytoplasm if the cell wall fails to contain the germling’s turgor pressure (29, 35–38). Nominally, septa allow for the free flow of cytoplasm between compartments (39, 40). However, if a germling suffers a cell wall rupturing injury, Woronin bodies migrate to the septal pore and serve to block cytoplasm flow from healthy hyphal compartments into the injured one, thereby saving the healthy septal compartments (31, 34, 41, 42).

Echinocandins, such as micafungin (MF), target active β-(1,3)-glucan synthesis (43, 44). For species that grow with a high degree of polarity (e.g., *Aspergillus* spp.), the echinocandin drug effect occurs primarily at the hyphal tip, where there is active β-(1,3)-glucan synthesis (5, 45). Non-apical septal compartments are protected from echinocandin wall stress by the separation septa provide between them and the more vulnerable apical compartments. Hence, echinocandins result in a fungistatic effect for *Aspergillus* spp. (5, 43, 45), as subapical compartments are largely unaffected (46).

Septation in filamentous fungi is regulated by the septation initiation network (SIN) (26, 30, 47, 48) (MEN in yeast and HIPPO in higher eukaryotes) (49, 50). Although the sources of all septation signals have not been mapped, one major component of SIN is the protein kinase SepH (24–26, 28, 30, 47, 51–55). When activated, SepH initiates septation through the kinase cascade SepH-SepL-SidB, where SepL and SidB are dependent on cofactors SepM and MobA, respectively (24, 25, 28, 47, 52, 53). SepH has several known upstream regulators. The cyclin-dependent protein kinase NimX regulates SepH through the small GTPase SpgA and its GTPase-activating proteins (GAPs) ByrA and BubA (24–26, 30, 47, 56). Negative regulators of SIN have also been reported. The protein kinase PomA has roles regulating cell polarity in *Schizosaccharomyces pombe (*57–60) and has been shown to be a negative regulator of SIN activity in *Aspergillus nidulans (*52). The cell cycle regulator, AnkA (59, 61–71), which is another protein kinase, has also been shown to repress SIN activity (52).

Strains lacking genes required to form septa have been shown to have significantly reduced viability compared to their wild-type counterparts when exposed to critical concentrations of cell wall-perturbing drugs (23–25). Control experiments carried out by Spence et al. in 2022 also determined that viability rates of non-septating strains were not affected by critical concentrations of fungicidal drugs that do not target the cell wall (23). These results imply that septa are critical for preserving vitality, specifically during cell wall stress.

These findings, in combination with evidence from our previous work, showing that SIN is activated by the CWI pathway in response to micafungin-induced cell wall stress (18), have led us to investigate this interaction. The CWI–SIN interaction could represent a critical adaptive mechanism that allows vegetative hyphae to rapidly compartmentalize cytoplasm in order to minimize losses under increased risk of cell wall failure. The potential of such a mechanism opens avenues for novel therapeutic strategies that could disable this adaptive feature when targeting the fungal cell wall (e.g., with echinocandins).

This work aims to elucidate the molecular dialogue between the CWI and SIN signaling pathways through genetic means. As *A. nidulans* has proven to be a productive model organism, due to its genetic tractability (72), its close relation to the pathogen *A. fumigatus* (73), and the fact that it features many conserved pathways, common among many other fungal species (74–78), we have chosen to conduct our studies using this model.

## RESULTS

### SIN is activated by micafungin-induced cell wall stress

**Fig 1:**
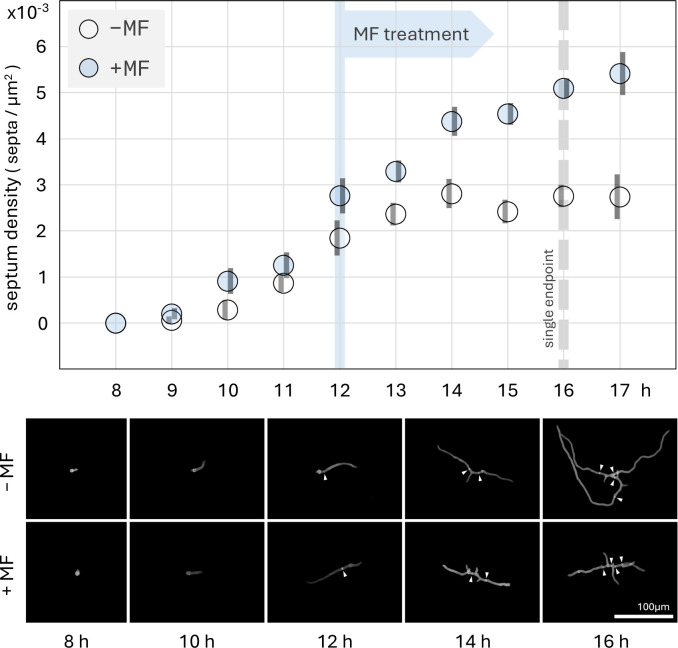
Top: time-course of the control strain (FGSC A1405) septum density from 8 to 17 h after coverslip inoculation. Freshly harvested conidia were adhered to coverslips using concanavalin A, and then submerged in liquid YGV growth media at 28°C. Micafungin (MF) was added for the shaded series 12 h after inoculation (blue flag) to a final concentration of 10 ng/mL. For each marker, N ≈ 90. Single-endpoint data for Fig. 2 were collected at the dashed line. Error bars are standard error. Bottom: selected images of germlings represented by plot data. White arrowheads highlight septa.

In 2020, Chelius et al. (18) found that under micafungin-induced cell wall stress, there was an increase in septum density (septa/µm^2^ growth area), and the SIN kinase SidB was differentially phosphorylated at three phosphorylation sites. We reproduced the septum density experiments for the wild-type control strain (FGSC A1405) (79) with more replicates, and the results have been summarized in Fig. 1.

**Fig 2:**
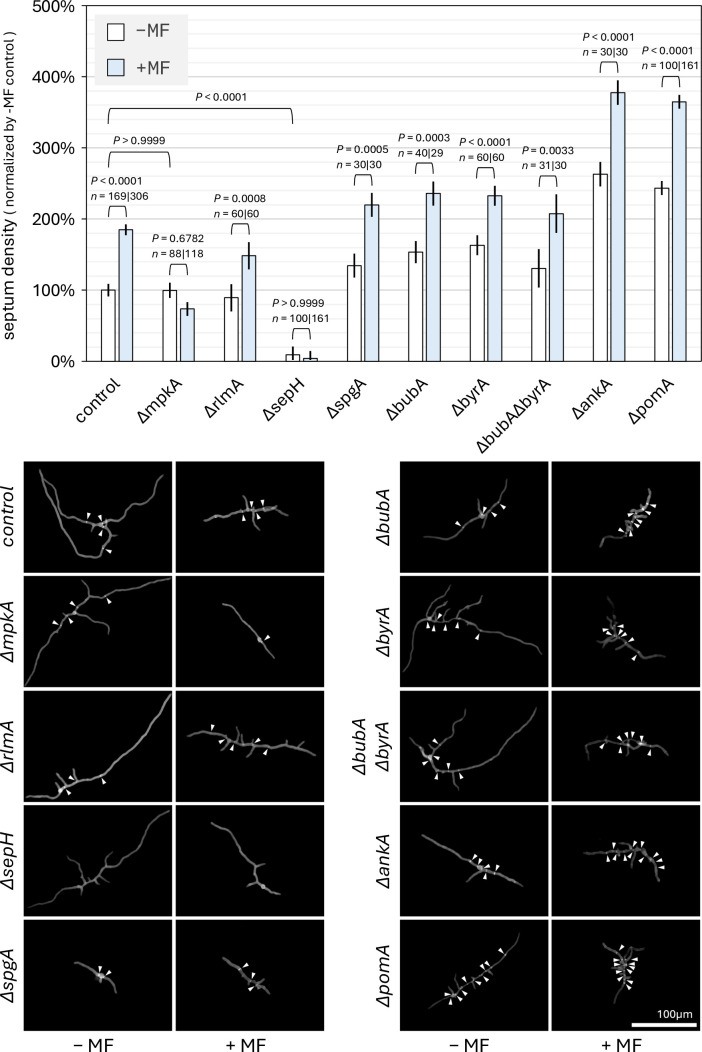
(Top) Normalized septum density 16 h after coverslip inoculation. For shaded bars, cell wall stress was induced with 10 ng/mL micafungin (MF) starting at hour 12. For each −/+ MF pair, the *P* value of the contrast is displayed along with the sample size of both bars. Error bars are standard error. (Bottom) Selected images represented by bar plot data. White arrowheads highlight septa. Control germlings from Fig. 1 are shown again for comparison with knockout germlings.

For the shaded series (+MF), we observed a dramatic increase in septum density starting when germlings were exposed to 10 ng/mL micafungin 12 h after inoculation, that continued until the end of the experiment at hour 17. This trend contrasts with the no-drug control (−MF), where septum density plateaued at about 0.00276 septa/µm^2^ approximately 13 h after inoculation. The trends diverged, such that at 16 h post-inoculation, the septum density of the drug-treated mycelia was 85% higher than that of the no-drug control (*P* < 0.0001) (Fig. 1 and 2).

### SIN is activated by CWI via MpkA

Our time-course experiments in Fig. 1 revealed that 16 h was the optimal timepoint to run statistical testing between phenotypes (see “Septum density coverslip assay,” below), so in Fig. 2, the remaining septum density measurements were made at the 16th hour.

We proceeded with an *∆mpkA* strain (FGSC A1404) (69, 79) to determine if MpkA, the terminal kinase of CWI, is required for the wild-type response to micafungin treatment. In Fig. 2, septum density is unresponsive to (i.e.*,* does not increase under) micafungin-induced wall stress for the *∆mpkA* strain (*P* = 0.6782). This implies that MpkA is essential to activate the SIN pathway under cell wall stress conditions. In the no-stress condition, the *∆mpkA* strain has comparable septum density to the control strain (*P* > 0.9999, Fig. 2), implying that MpkA is removed from septation regulation in the absence of wall stress. These results highlight that CWI, rather than a parallel upstream pathway, is responsible for SIN activation in response to cell wall stress.

To determine whether crosstalk occurs downstream of CWI through the transcription factor RlmA, an *∆rlmA* strain was constructed. Septum density for the *∆rlmA* strain was 66.3% higher under cell wall stress than no stress (*P* = 0.0008, Fig. 2), implying that the crosstalk signal is not transduced transcriptionally through RlmA regulation and exits CWI through MpkA.

### SIN receives CWI signal through SepH

To identify the entry point of the CWI signal into the SIN pathway, we used a genetic approach and deleted known SIN signaling proteins systematically. We began our investigation with the protein kinase SepH.

In the absence of SepH, septation is significantly reduced, with septum density at 9.3% that of the control strain in the no-stress condition (*P* < 0.0001, Fig. 2). This finding is consistent with literature showing that SepH plays a role in the assembly of the septation initiation complex at the spindle pole body (26, 28, 51, 55). Furthermore, no change in septum density is observed under micafungin-induced cell wall stress conditions in the *∆sepH* mutant (*P* > 0.9999, Fig. 2). These results suggest that SepH is essential for transducing the CWI signal to the SIN pathway, and that the signal must enter SIN at or upstream of SepH.

We identified five upstream regulators of SepH in the literature: a small G-protein, SpgA (26, 52), its two GAPs, BubA and ByrA (30, 47), and two protein kinases, AnkA and PomA (52). To determine if any of these SIN regulators receive the CWI stress signal, we systematically knocked out the corresponding protein-coding genes of each regulator and measured septum density with and without micafungin treatment. Additionally, we created and tested a double knockout of the two GAPs that regulate SpgA in parallel to rule out the possibility that the GAPs transduce redundant CWI signals.

In Fig. 2, for each of the knockouts *∆spgA*, *∆bubA*, *∆byrA*, *∆bubA∆byrA*, *∆ankA*, and *∆pomA*, the wild-type response is observed, where septum density is higher under cell wall stress conditions compared to the unstressed condition (all *P* ≤ 0.0033). As no proteins upstream of SepH in the SIN pathway were found to be essential for signal transduction, we propose that SepH serves as the entry point of the CWI stress signal into the SIN pathway.

## DISCUSSION

In this work, we found that the SIN pathway responds to cell wall stress by increasing septum density. Using a genetic approach, we discovered that the septation response is mediated by a crosstalk signal, where CWI activates SIN during micafungin-induced wall stress.

**Fig 3:**
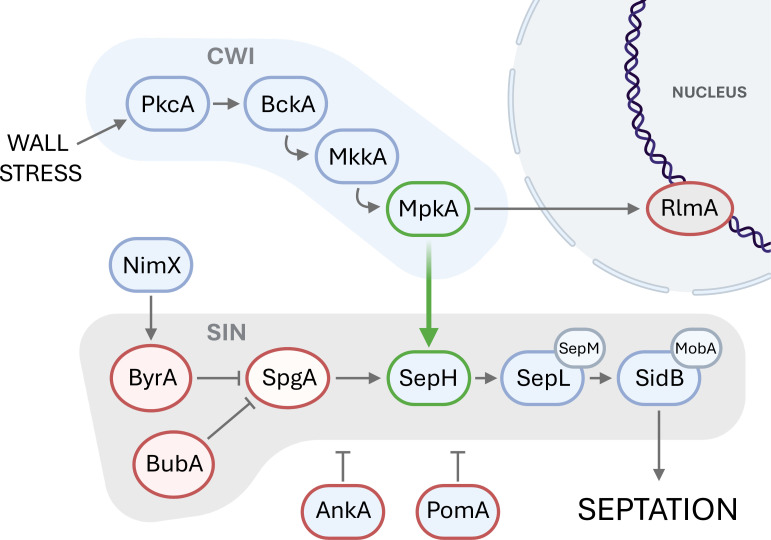
Signal diagram of the cell wall integrity (CWI) and septation initiation network (SIN) pathways. Red border = knockout strain had a wild-type response to wall stress (increased septum density). Green border = knockout strain did not change septum density in response to wall stress. Our results suggest that the crosstalk is transduced between MpkA (CWI) and SepH (SIN) kinases under cell wall stress conditions, as highlighted by the green arrow. For the proteins in this figure, orthologous names of other common ascomycete models are listed in Table 1.

More specifically, we discovered that the stress signal is transmitted from CWI by the MAP kinase MpkA and is received in SIN by the protein kinase SepH. Nominally, septum formation is regulated through the cyclin-dependent kinase (CDK) NimX and is thereby coupled to mitosis and the larger cell cycle (26, 80–83). Our results demonstrate that septum formation can be propagated independently by stress-induced CWI signaling through MpkA. In this mapping, SIN is shared by both processes, with SepH serving as the junction as shown in Fig. 3.

We hypothesize that upregulation of septation through CWI–SIN crosstalk is an adaptive response that preserves hyphal vitality during cell wall stress. Germlings with compromised cell wall integrity are at a higher risk of losing septal compartments to hyphal injury. Septal plugging nominally isolates cytoplasmic loss to a single septal compartment, so construction of additional septa manages heightened risk by reducing average compartment volume through subdivision. For echinocandin stress, apical compartments are most at risk due to inhibition of β-(1,3)-glucan synthase, which is active primarily at hyphal tips (5, 45). Should an apical compartment be lost, increased septum density reduces the distance from the surviving hypha to the pre-loss colonization front. This shorter distance expedites recovery, conserves resources needed to restore growth, and bolsters germling vitality in the colonization of its environment.

Our hypothesis is supported by findings in *A. nidulans* and *A. fumigatus*, demonstrating that strains with compromised septation are more sensitive to cell wall stress. Specifically, hypo-septator (HypoS) strains (strains with innately reduced septum densities, e.g*., ∆bud3, ∆bud4, ∆rho4,* and *∆sepL*)*,* non-responding septator (NRS) strains (strains that do not upregulate septation under wall stress, e.g*., ∆mpkA*), and strains that fall into both classes (e.g*., ∆sepH*) are significantly more sensitive to cell wall stress than their isogenic control strains (18, 23–25). In Fig. S1, we confirmed in a conidium viability assay, that conidia of HypoS and NRS strains in our study (*∆sepH, ∆sepL,* and *∆mpkA*) are more sensitive to cell wall stress than control conidia (all *P* ≤ 0.0032). In contrast, conidia of strains that were neither HypoS nor NRS (*∆rlmA, ∆spgA, ∆bubA, ∆byrA, ∆bubA∆byrA, ∆ankA, and ∆pomA*) formed colonies at rates comparable to the control (all *P* ≥ 0.3515), or, for *∆pomA,* possibly higher than that of the control (*P* = 0.0829).

In the septum density single-endpoint experiments (Fig. 2), we observed that micafungin inhibits growth for a number of strains, including the control, *∆mpkA*, and *∆sepH* strains. This could be due to the inhibition of β-(1,3)-glucan synthase; however, we hypothesize that this is an active stress-triggered response through an unknown mechanism. In Fig. S2, growth area (µm^2^) is plotted over time, similar to septum density in Fig. 1. Here, control and *∆sepH* germlings continue attenuated growth after exposure to micafungin. They then slow growth to a pause 3–4 h post-exposure. This abrupt slowing, and then pause of growth, hints at an active regulatory mechanism. Such a mechanism would remove germlings from risk associated with tip extension under compromised β-(1,3)-glucan synthase activity, preventing cell wall instability at the hyphal tip.

The attenuated growth observed in this timeframe is also strong evidence that germlings manage to survive at 10 ng/mL micafungin exposure. This increases our confidence that the NRS phenotype observed for *∆mpkA* and *∆sepH* in Fig. 2 is not due to a lack of survival under micafungin exposure. To that end, observations during our coverslip experiments further corroborate this claim. In the coverslip procedure, germlings were exposed to micafungin starting 12 h after inoculation and were not fixed for imaging until hour 16. Germlings that were unable to survive had ample time to deteriorate during the 4 h exposure period. Upon imaging, dead germlings that had not completely deteriorated were either shriveled up, folded unnaturally, or broken in two. We did not include these in our studies, as we were only interested in the phenotypes of germlings surviving at the critical concentration (10 ng/mL) of micafungin.

To rule out the possibility that RlmA contributes significantly to CWI–SIN crosstalk transcriptionally, we constructed an *∆rlmA* strain in the same genetic background. After running the coverslip septum density assay (Fig. 2), we found that septation in *∆rlmA* responds nominally to micafungin stress, implying that RlmA does not contribute significantly to CWI–SIN crosstalk. In the conidium viability assay, we found that micafungin treatment of *∆rlmA* did not result in reduced viability under wall stress (*P* = 0.7250) (Fig. S1), further implying that transcription plays a diminished role in responding to echinocandin stress.

Our findings in *A. nidulans* have been strikingly consistent with those reported in *A. fumigatus*. That is, deletion mutants of SIN orthologues *sepH* and *sepL* are significantly more sensitive to echinocandin wall stress than their control strains (23, 24), whereas those of SIN orthologues *spgA, bubA,* and *byrA* are not in both *A. fumigatus (*24, 25) and *A. nidulans* (23). Septation is also completely eliminated or considerably reduced for *∆sepL* (Fig. S3) and *∆sepH* deletions, respectively, in both species, whereas it is not for knockouts of other SIN regulators (23, 24). These consistencies suggest that the septation response to wall stress is at least conserved broadly across Eurotiales. Furthermore, conservation of CWI and SIN pathway components across a wide range of fungal species (19, 26, 48, 73, 84–88) raises the possibility that the crosstalk mechanism discovered in this study may, in some semblance, be employed by fungi more broadly to respond to cell wall stress.

To the best of our knowledge, our discovery represents the first time CWI–SIN crosstalk has been described in filamentous ascomycetes and may be applicable to fungi more broadly. Comparative genomic and functional analyses of CWI–SIN crosstalk across more diverse fungal species could shed brighter light on its evolutionary origins and functional significance. In particular, the septation response to cell wall stress is likely relevant to clinically significant fungal pathogens. Echinocandins target cell wall synthesis and are widely used to treat mycoses (5, 44, 89). Their efficacy is species specific though (5, 43), which our results suggest may be due to the CWI–SIN response and how it preserves mycelial vitality during echinocandin stress. The discovery of CWI–SIN crosstalk advances the molecular understanding of echinocandin pharmacology, which we hope will contribute to the development of novel fungal treatment strategies to overcome fungistatic limitations of the echinocandin drug class for filamentous fungi.

## MATERIALS AND METHODS

### Strains

A full list of strains used in this study is available in Data Sheet S1.

Our experiments were carried out using strains in the SO451 (FGSC A1166) background (*pyrG89; wA3; argB2; ΔnkuA^ku70^::argB pyroA4; sE15 nirA14 chaA1 fwA1*) (69, 79, 90). Several of the kinase knockout strains had already been generated in the Kinase Knockout Library (KKOL) by De Souza et al. in 2013 (69). These strains (*∆mpkA, ∆sepH, ∆sepL, and ∆pomA*) were acquired from FGSC (strain numbers A1404, A1334, A1306, and A1371, respectively) (69, 79). We used the KKOL wild-type control strain (FGSC A1405) as the control for our experiments that has *pyrG* transformed back into SO451 (69). The remaining knockout strains were SO451 transformants generated using CRISPR-Cas9 and homology directed repair (HDR) workflow.

### Storage and preparation

Fresh conidia were used in all experiments. Fungal strains were stored in phospho-buffered 20% glycerol stocks at −80°C. Fresh conidia suspensions were produced by growing banked conidia on MAGVS solid media (2% glucose, 2% malt extract, 0.2% peptone, 1.5% agar, 0.95 M sucrose, with Vitamins and Hutner’s Trace Elements supplements) (79). Conidia were harvested from the MAGVS plates using DI water and separated from mycelia and conidiophores with vigorous agitation and subsequent filtration through glass wool. Conidium concentrations were measured under microscope with a hemocytometer.

### Septum density coverslip assay

Conidia were adhered to coverslips for the duration of the time-course study with concanavalin A (conA) (Thermo Fisher J61221). Unless stated otherwise, for all of the steps of the procedure, the same side of the coverslip was always facing in the upward direction to ensure that all of the treatments were performed to the same side of the coverslip. Square coverslips (no. 2, 25 mm) were dusted off with compressed air, then autoclaved. Sterile coverslips were then submerged in a conA solution (100 µL 132 mM CaCl_2_, 10.7 mg conA in 20 mL PBS) for 30 min at 30°C. They were then dipped in DI water three times and placed on Teflon squares inside of a sterile petri dish to dry overnight.

Conidia were adhered to the coverslips by pipetting 1 mL of a 5  ×  10^4^ conidia/mL suspension directly onto the surface of each coverslip. Excess conidium suspension was pipetted up, leaving the coverslips damp. The conidia were left to adhere to the coverslips for 1 h at 30°C. The coverslips were then removed from their Teflon squares and submerged in sterile petri dishes filled with 25 mL pre-heated liquid YGV media (0.5% yeast extract, 2% glucose, with Vitamins and Hutner’s Trace Elements supplements) (79). The petri dishes were then placed in an oven-style incubator set to 28°C. For the micafungin stress condition, 25 µL of 10 µg/mL micafungin in DI water was added to the petri dish 12 h after the start of incubation (10 ng/mL final conc). Micafungin was mixed into the media by gently pipetting the media up and down with a 1000 µL pipette.

Enough coverslips were prepared so that one coverslip per timepoint could be harvested for imaging. At each timepoint, a coverslip was removed from the liquid media and submerged for at least 30 min in a petri dish containing filtered fixative (3.7% paraformaldehyde, 0.2% Triton X-100, 50 mM NaH_2_PO_4_, KOH flakes until clear, pH to 7.0 with HCl). After fixing, a microscope slide cleaned with 95% EtOH and 12 µL staining solution (1 mg/mL calcofluor white, 0.5 mg/mL Evan’s blue solution mixed 1:1 with glycerol, filtered) was pipetted onto the middle of the slide. A coverslip was then removed from the fixative dish, blotting excess fixative onto a sterile Kimwipe and placed face down on the slide over the drop of stain. Gentle pressure was used to flatten the coverslip and mycelia onto the slide.

Approximately 30 germlings were imaged for each timepoint. Growth area (pixels), number of tips, number of germ tubes, and number of septa were recorded for each germling. Images were taken using an Olympus IX81 microscope equipped with a fluorescent Lumencor SOLA Light Engine and Hamamatsu ORCA-spark camera. Images were cropped, and extraneous germlings were removed for clarity only in the lower multi-panels of Fig. 1 and 2. Raw, unmodified images were used in data collection. Growth area in pixels was measured using ImageJ’s automatic threshold feature. ImageJ measurements were validated by eye visually and taken with as little manual intervention as possible. Growth area was converted from pixels to µm^2^ using a hemocytometer to calibrate the conversion.

In our time-course study, we found that the 16th hour timepoint offered the maximum difference in septum density between the drug and no-drug treatments without a drop-off in in the number of germlings measured per coverslip. At timepoints beyond 16 h, the germlings became increasingly large and interwoven, leading to a decrease in the number of suitable germlings available to image per timepoint and a bias toward smaller individuals. Having established the 16th hour as the optimal timepoint for analysis, we transitioned to a single-endpoint strategy for the knockout strains, allowing us to compare treatments independent of time. So, for each treatment, we measured septum density 16 h after inoculation. To facilitate comparison in this study, we normalized single-endpoint septum density measurements to the mean no-drug control strain density at the 16th hour timepoint (0.002758 septa/µm^2^).

### Significance testing

For septum density data in Fig. 1 and 2, standard error bars and *P* values were calculated with a one-way analysis of variance table of a mixed-effects model (91). To account for variation between experimental batches, the day each datapoint was collected was considered to be a random-effect in the mixed-effects model. The analysis was run in R (92) using the car (93)*,* lme4 (91)*,* and emmeans (94) libraries.

### Conidium viability assay

Control medium (CM) and micafungin (MF) medium plates were made using MAGV solid medium (2% Glucose, 2% Malt Extract, 0.2% Peptone, 1.5% Agar, with Vitamins and Hutner’s Trace Elements supplements) (79). For MF plates, after the molten MAGV medium had cooled to below 60°C, micafungin was added to a final concentration of 7 ng/mL. Media were then poured into 100 mm petri dishes and allowed to solidify.

For each strain (e.g., control, *∆mpkA*, *∆sepH*, etc.), a suspension of conidia was harvested and prepared as described in *“*Storage and preparation,” above. The suspensions were diluted with DI water to 2  ×  10^3^ conidia/mL, and then 50–100 µL (strain-dependent) was spread onto each plate. For each strain, the same suspension and volume was used to inoculate four replicate CM and MF plates. The plates were then incubated at 28°C for 2 days or until colonies were clearly formed, after which colonies on each plate were counted.

To calculate percent conidium viability (*P_v_*) under MF stress for a given mutant strain, the number of colonies on each MF plate was divided by the average number of colonies on the CM plates, yielding a *P_v_* value for each MF plate. Each *P_v_* was then normalized through division by the average *P_v_* of the control strain on MF. The final result was then given by the mean and standard error of the normalized *P_v_* values for each mutant strain.

### CRISPR-Cas9 transformation procedure

Our transformation protocol was adapted from the protoplasting procedure described in the 2012 study of Oakley et al. (95) and the CRISPR-Cas9 mediated transformation procedure described in the 2020 study of van Rhijn et al. (96).

Transformation-ready protoplasts were obtained similar to Oakley et al.’s study (95) using VinoTaste Pro enzyme (Novozymes). The concentration of the washed protoplast suspension was measured using a hemocytometer and either diluted with buffer or concentrated by centrifugation to 5  ×  10^6^ protoplasts/mL.

The CRISPR/Cas9 ribonucleoprotein (RNP) complex was prepared immediately before transformation by adding (in order): 22 µL HEPES buffer (20 mM HEPES free acid, 150 mM KCl, pH to 7.5 with KOH), 0.62 µL Cas9 dilution buffer (supplied with Cas9 Plus enzyme), 0.88 µL of 1.7 µg/µL Cas9 Plus (Sigma-Aldrich CAS9PL), 2 µL duplex buffer (IDT 11–01-03-01), 0.5 µL of 100 µM 5p sgRNA, and 0.5 µL of 100 µL 3p sgRNA. The solution was vortexed, spun, and allowed to incubate at room temperature for 5 min.

The transformation mixture was assembled by adding (in order) to the assembled RNP complex: 200 µL of 5 × 10^6^/mL protoplast suspension, 2–10 µg linear DNA repair cassette (max vol: 50 µL), and 25 µL 60% PEG solution (60% w/v PEG 3350, 50 mM CaCl_2_, 450 mM Tris–HCl, pH 7.5 with KOH). The mixture was gently vortexed and incubated on ice for 50 min. At 10–15 min intervals, the mixture was gently vortexed to maintain homogeneity. Sequences of CRISPR sgRNAs and deletion cassette oligos can be found in Data Sheet S2.

Membrane permeation was achieved through heat shock. After 50 min of incubation on ice, 1.2 mL warm (~45°C) 60% PEG solution was added to the transformation mixture and rapidly mixed by pipetting up and down, and then by gentle vortexing. The mixture was allowed to incubate for 20 min at room temperature, vortexing at 10 min intervals to maintain homogeneity.

Plates with MM + VS selective media (1% glucose, 1.5% agar, 0.05% MgSO_4_, 0.95 M sucrose, 0.6% NaNO3, 0.152% KH_2_PO_4_, 0.052% KCl, with vitamins and Hutner’s trace elements supplements) (79) were each inoculated with 50 µL transformation mixture and incubated at 28°C until conidiation was observed. Colonies were then picked and re-plated for genotyping by diagnostic PCR (Data Sheet S3; Fig. S4).

## Data Availability

Data presented in this work are available in the figures and supplemental files. Strains used in this study have been retained and are available from FGSC (79) and/or upon request to the corresponding author.

## AFTER EPUB

*[This article was published on 13 December 2024 with the incorrect Data Sheet S3. The supplemental material was corrected in the current version, posted on 3 January 2025.]*

## SUPPLEMENTAL MATERIAL

Data Sheet S1 msphere.00641-24-s0001.xlsx Detailed list of *A. nidulans* strains used in this study.

Data Sheet S2 msphere.00641-24-s0002.xlsx Oligonucleotide sequences used to generate recombinant strains.

Data Sheet S3 msphere.00641-24-s0003.xlsx Diagnostic primers used to confirm recombinant strain genotypes.

Supplemental Materials msphere.00641-24-s0004.pdf Data sheet captions and supplemental figures.

ASM does not own the copyrights to Supplemental Material that may be linked to, or accessed through, an article. The authors have granted ASM a non-exclusive, world-wide license to publish the Supplemental Material files. Please contact the corresponding author directly for reuse.
