# Supplementary material for: Protein kinases MpkA and SepH transduce crosstalk between CWI and SIN pathways to activate protective hyphal septation under echinocandin cell wall stress
Source: mSphere. 2024 Dec 13;10(1):e00641-24. doi: 10.1128/msphere.00641-24 (PMC11774030; doi:10.1128/msphere.00641-24)
Supplement: Supplemental Materials — Data sheet captions and supplemental figures. [file msphere.00641-24-s0004.pdf]

SUPPLEMENTAL MATERIALS

Data Sheet S1. Detailed list of *A. nidulans* strains used in this study.

Data Sheet S2. Oligonucleotide sequences used to generate recombinant strains.

Data Sheet S3. Diagnostic primers used to confirm recombinant strain genotypes.

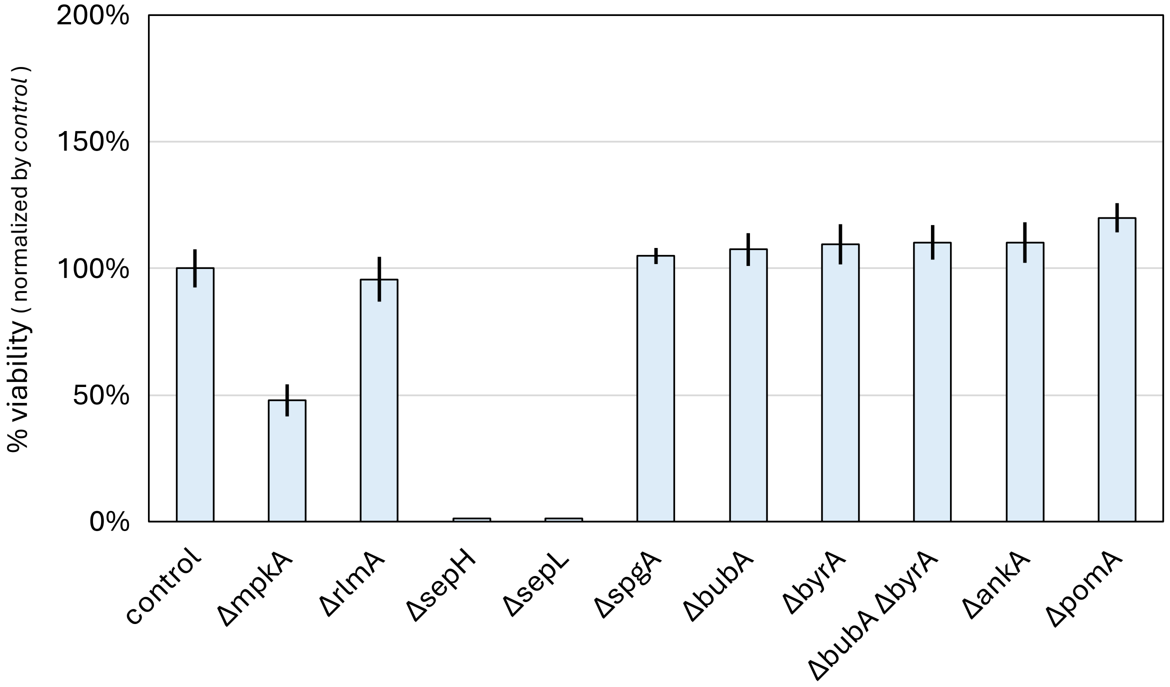

Figure S1. Percent conidium viability of SIN knockout strains under 7 ng/mL micafungin stress on MAGV plates. Error bars are standard error. No *ΔsepH* or *ΔsepL* colonies were observed.

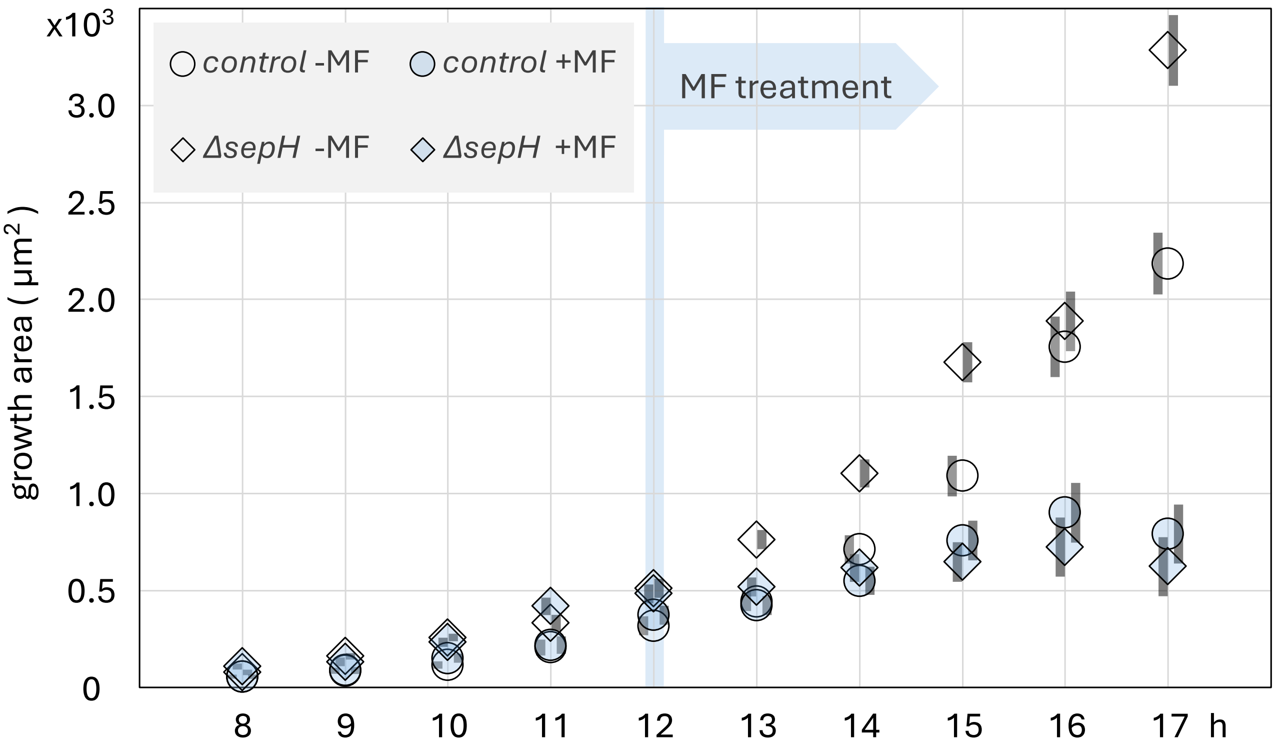

Figure S2. Growth area timecourse of *control* and *ΔsepH* strains, with and without 10 ng/mL micafungin (MF) treatment starting at hour 12. From hour 12 to 15, micafungin treated strains grow slowly in area for 3 h before pausing growth from hour 15 to 17.

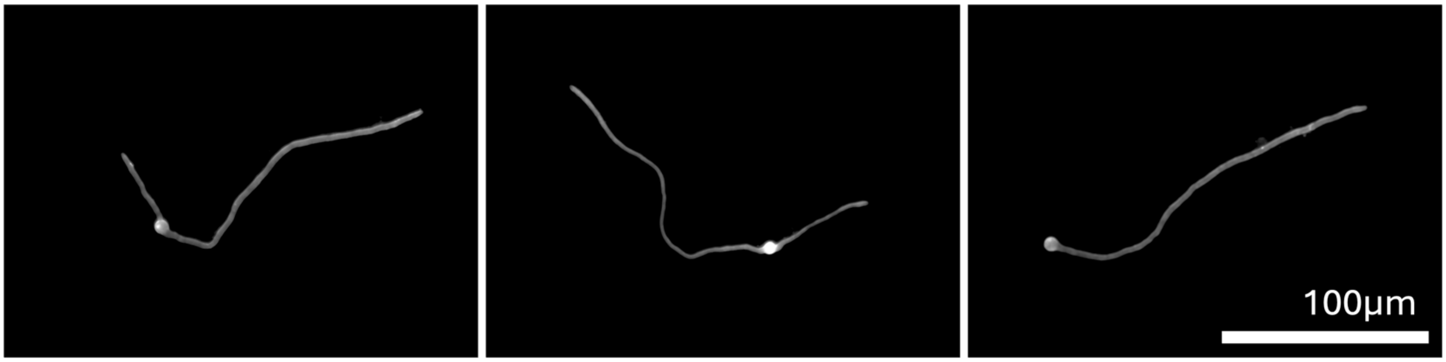

Figure S3. Microscopy images showing *ΔsepL* strain with no septa. Images were taken 16 h after inoculation similarly to -MF images in Figure 2.

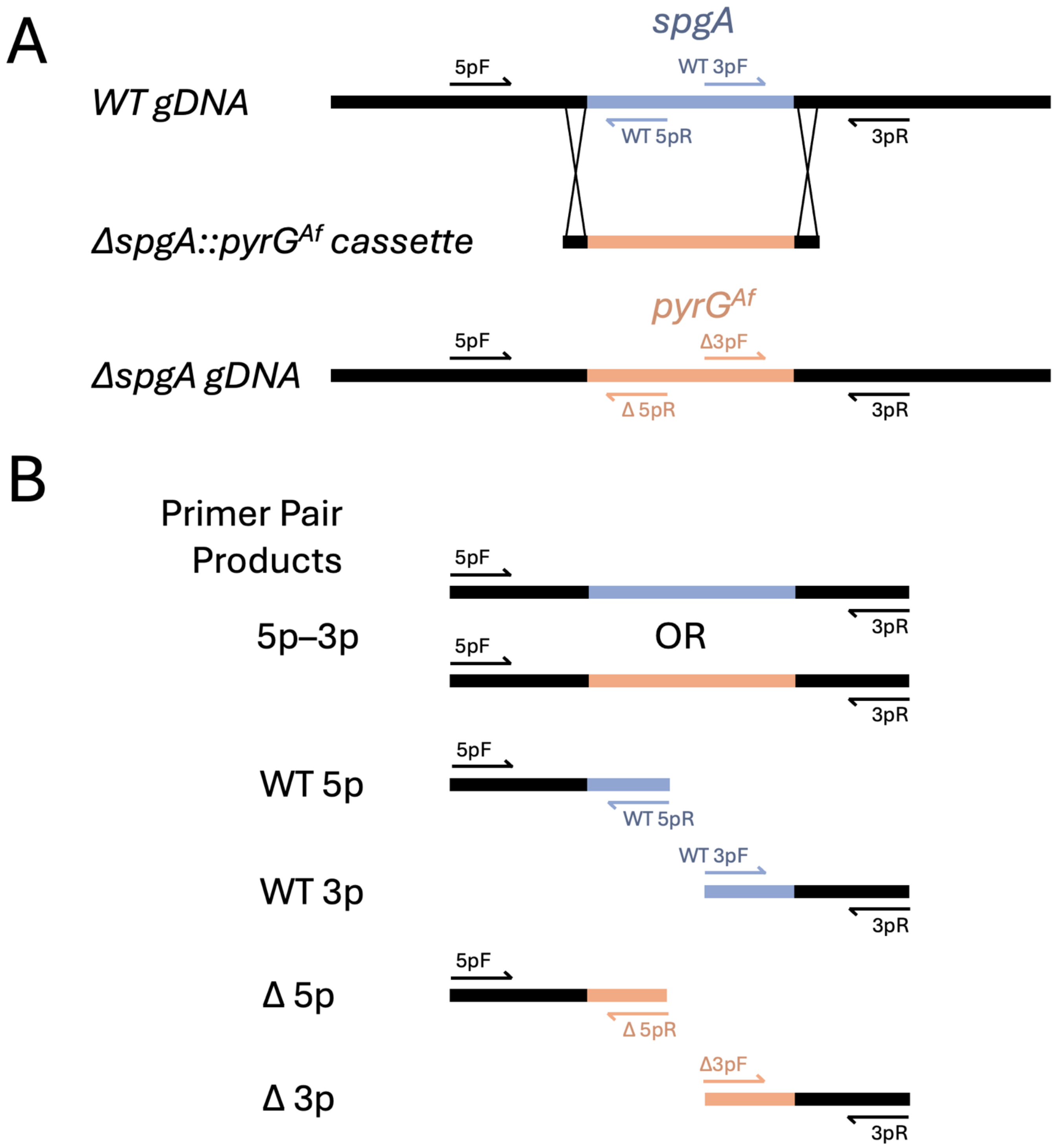

Figure S4-1. An overview of our diagnostic PCR (DPCR) genotype verification strategy. (A) We generated knockout strains using a Cas9-mediated homology directed repair (HDR) technique, where we replaced the target gene (i.e. *spgA*) with a HDR cassette containing an auxotrophic selection marker (*pyrG* or *pyroA*). Cas9 cut sites were engineered to remove as much of the *wild-type* (*WT)* gene as possible, with one near the 5-prime (5p) end and a second near the 3-prime (3p) end of the gene. The repair cassette included approximately 50 BP of homology on both ends, complementary to the *WT* sequence outside of the Cas9 cut sites, facilitating targeted insertion of the cassette. (B) For each transformation, we designed five primer pair products (using six primers total) to confirm that the desired genotype was obtained. The first product (5p-3p) spans the entire gene/knockout region, with primers 5pF upstream and 3pR downstream of the repair site. This produces a DPCR product that varies in length, depending on whether *WT* or *knockout* gDNA is present. The second and third DPCR products (WT 5p and WT 3p) appear only if the *WT* gene is present and, likewise, the fourth and fifth DPCR products (Δ 5p and Δ 3p) appear only if the HDR cassette has replaced the *WT* gene. Because these four DPCR products use either the 5pF or 3pR primer for one side of each product, they span both inside and outside the HDR repair site, serving to confirm that both sides of the repair template was (or was not) inserted correctly.

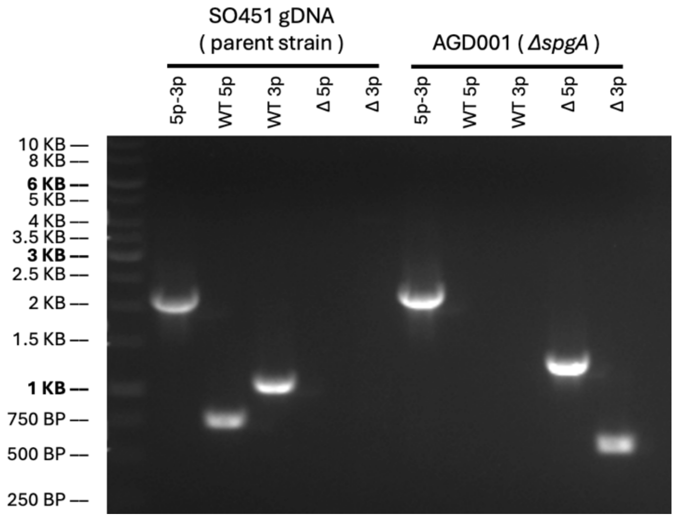

Figure S4-2. Diagnostic PCR gels of AGD001 (*ΔspgA* strain) gDNA, confirming *ΔspgA::pyrG* genotype in SO451 background.

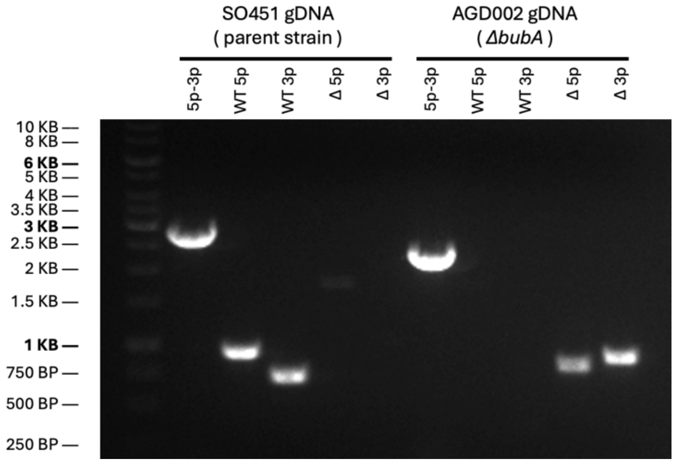

Figure S4-3. Diagnostic PCR gels of AGD002 (*ΔbubA* strain) gDNA, confirming *ΔbubA::pyrG* genotype in SO451 background.

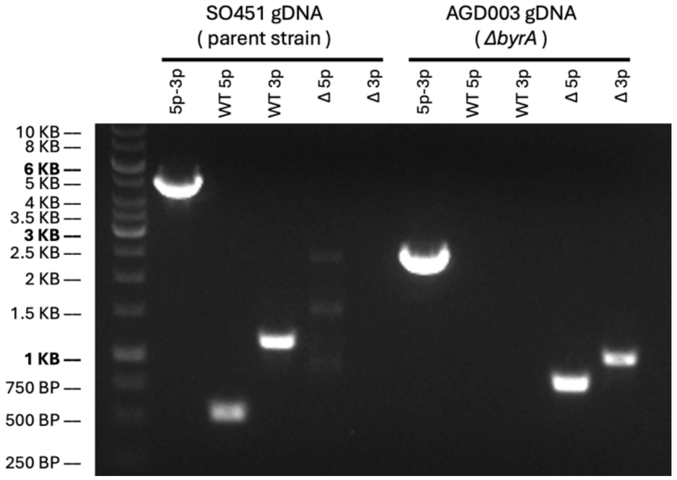

Figure S4-4. Diagnostic PCR gels of AGD003 (*ΔbyrA* strain) gDNA, confirming *ΔbyrA::pyrG* genotype in SO451 background.

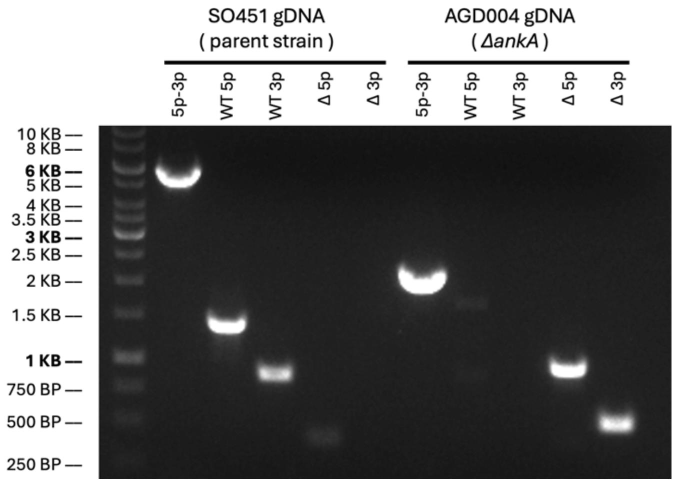

Figure S4-5. Diagnostic PCR gels of AGD004 (*ΔankA* strain) gDNA, confirming *ΔankA::pyrG* genotype in SO451 background.

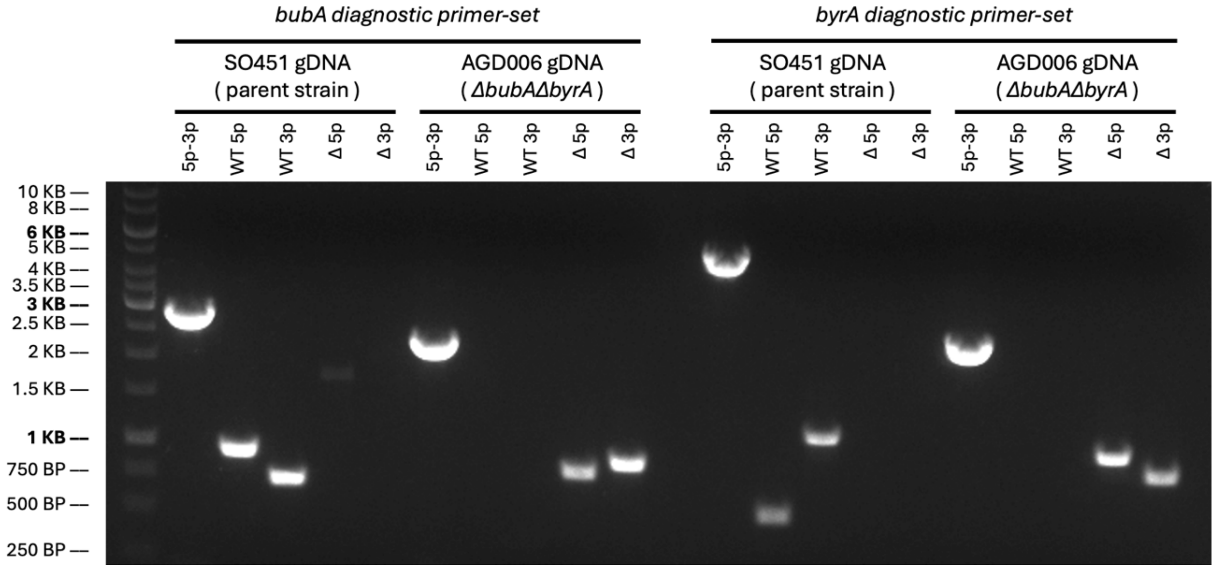

Figure S4-6. Diagnostic PCR gels of AGD006 (*ΔbubAΔbyrA* double deletion strain) gDNA, confirming *ΔbubA::pyrG ΔbyrA::pyroA* genotype in SO451 background.

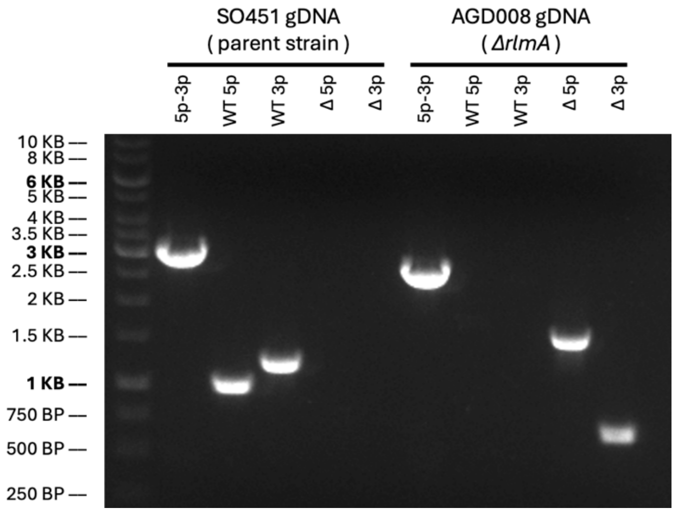

Figure S4-7. Diagnostic PCR gels of AGD008 (*ΔrlmA* strain) gDNA, confirming *ΔrlmA::pyrG* genotype in SO451 background.
